# Microstructure Evolution and Mechanical Response of a Direct Quenched and Partitioned Steel at Different Finishing Rolling Temperatures

**DOI:** 10.3390/ma16093575

**Published:** 2023-05-06

**Authors:** Yajun Liu, Xiaolong Gan, Wen Liang, Guang Xu, Jianghua Qi, Man Liu

**Affiliations:** 1 The State Key Laboratory of Refractories and Metallurgy, Key Laboratory for Ferrous Metallurgy and Resources Utilization of Ministry of Education, Wuhan University of Science and Technology, Wuhan 430081, China; 2 Technical Center, Hunan Valin Lianyuan Iron and Steel Co., Ltd., Loudi 417009, China

**Keywords:** finishing rolling temperature, direct quenched and partitioned steel, residual austenite, grain size, lath martensite

## Abstract

The effects of finishing rolling temperature on the microstructure and mechanical properties of a direct quenched and partitioned (DQ&P) steel were investigated by a thermal simulation machine, a field emission scanning electron microscope (FE-SEM), electron backscattering diffraction (EBSD), and a transmission electron microscope (TEM). The results show that the original austenite grain size was refined by 31% as the finishing rolling temperature decreased from 920 °C to 840 °C, leading to the formation of the finest martensite lath at 840 °C. At the same time, the lower finishing rolling temperature resulted in a higher dislocation density, and consequently improved the stability of the retained austenite. Moreover, compared to the conventional Q&P process, the comprehensive mechanical properties of a steel with similar chemical composition can be enhanced by DQ&P processing. With the decrease of finishing rolling temperature from 920 °C to 840 °C, the strength and total elongation increases. The yield strength, tensile strength, and total elongation reach the maximum values of 1121 MPa, 1134 MPa, and 11.7%, respectively, at 840 °C.

## 1. Introduction

Quenching and partitioning (Q&P) steel is one of the new-generation advanced high-strength steels with excellent comprehensive properties. Its microstructure usually consists of martensite and retained austenite (RA) [1,2,3]. Normally, Q&P steel is quenched to a quench stop temperature between martensite starting temperature (Ms) and finishing temperature (Mf) after full or partial austenitizing, and then held isothermally in that temperature (one-step) or reheated to a somewhat higher temperature (two-step) for carbon partitioning [4,5]. Recently, a modified approach to conventional Q&P processing is the direct quenching and partitioning (DQ&P) route, in which thermomechanical rolling is followed by direct quenching to a pre-determined temperature between Ms and Mf and subsequently proceeding to a carbon partitioning process. This approach has attracted much attention [6,7]. Compared to conventional Q&P processing, the DQ&P process involves no additional reheating, but rather quenching and partitioning directly after hot rolling [8,9]. On the basis of Q&P steel, Thomas et al. proposed a non-isothermal partitioning process to directly produce a Q&P microstructure by hot rolling, which proves that the DQ&P process is a feasible processing method for fabricating high-strength steel [8]. Jirková et al. adopted a direct Q&P process after hot deformation to refine grains and improve mechanical properties [9].

In other words, the DQ&P process is more practical in industrial production. The DQ&P processing is not only intended to make the process more environmentally friendly, i.e., more energy efficient, but also more technically feasible in industrial practice. [10,11,12]. Liu et al. used the direct quenching and partitioning process after hot forming to generate a two-way structure composing of ultrafine-grained retained austenite and martensite, leading to an increase of the total elongation from 6.6% to 14.8% and the improvement of the mechanical properties, i.e., the product of strength and elongation is reached at 22.4 GPa·% [13]. The formation of equiaxed fine grains caused by deformation at 1000 °C improves the stability of austenite during partitioning [6]. Mahesh et al. obtained a structural steel with a yield strength larger than 1100 MPa through the direct Q&P process, which also presents better ductility and impact properties than that of low carbon steel at the same strength grade [14].

Thermomechanical controlled processing (TMCP) typically involves a two-stage rolling process: first a recrystallizing rough roll above the recrystallization temperature, followed by a finishing roll in the non-recrystallizing zone below the recrystallization stop temperature to achieve fine, lumpy grains in the structure. The Q&P process together with the thermomechanical control process (TMCP) could effectively improve the overall mechanical properties of steels by refined martensite [15]. The martensite transformation is diffusionless and mainly influenced by chemical compositions and a state of austenite. When the austenite grain size is small, the matrix strength is higher, and the martensite transformation would start at a lower temperature, resulting in less martensite during quenching. In this case, the available carbon atoms are reduced during partitioning, and the secondary martensite transformation during the final cooling stage tends to occur due to the less stable metastable austenite. Therefore, the control of austenite grain size is critical. The finishing rolling temperature can directly determine the austenite grain size of the parent phase, and thus it can affect the volume fraction and stability of RA in the microstructure, which in turn influences the overall performance of the high-strength steels. Therefore, it is of interest to study microstructure evolution and mechanical behavior at different finishing rolling temperatures of a DQ&P steel, which provides the reference for designing TMCP parameters in industrial production.

## 2. Experimental Material and Method

**Figure 1:**
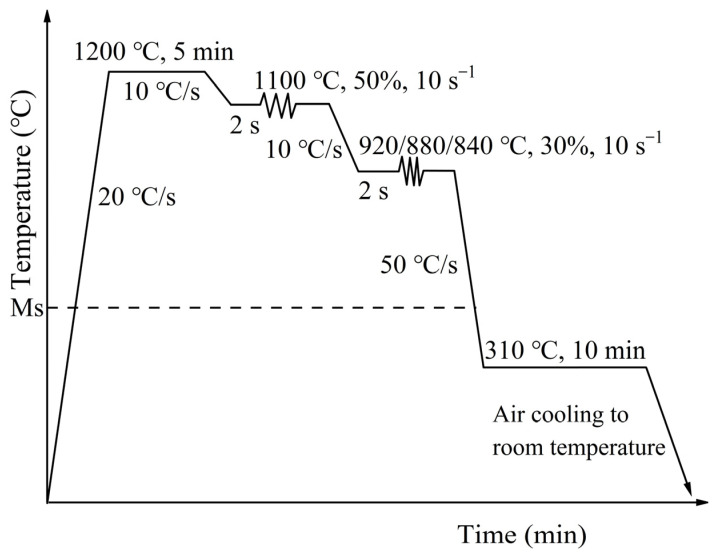
Schematic of thermal simulation experimental process.

The experimental material was taken from the steel cast slab produced in a steel plant with the chemical composition shown in Table 1. Firstly, the experimental material was cut into 250 × 100 × 60 mm^3^ billets. The samples were heated to 1200 °C and in isothermal holding for 4 h at nitrogen atmosphere to ensure the homogenous distribution of microalloying elements in the steel, then followed by water-cooling to room temperature. The specimens of Φ8 × 16 mm for hot simulation were prepared from the austenitized rectangle samples. The simulation experiments were conducted on a Gleeble-1500D thermal simulation tester (DSI, Nashville, TN, USA). The Ms and Mf of the tested steel were calculated by JMatPro 7.0 software (Sente Software Ltd., Guildford, UK) to be 349.4 °C and 234.1 °C, respectively. Hence, the quenching temperature of 310 °C was selected during DQ&P treatment. The processing routes are shown in Figure 1. Firstly, the specimens were heated to 1200 °C at 20 °C/s and kept for 5 min for austenitization followed by cooling to 1100 °C at a cooling rate of 10 °C/s. For the simulated roughing rolling process, the 50% strain with a strain rate of 10 s^−1^ was applied after holding for 2 s at 1100 °C. Then, specimens were all cooled to the pre-determined simulated finishing rolling temperature at a cooling rate of 10 °C/s. The 30% strain at a strain rate of 10 s^−1^ after holding for 2 s at simulated finishing rolling temperatures of 920 °C, 880 °C, and 840 °C was utilized. The strain of 50% at 1100 °C and 30% at finishing rolling process were utilized to reach the maximum deformation range of the two-step deformation in thermal simulation experiment. Afterwards, the deformed specimens were cooled to 310 °C at a cooling rate of 50 °C/s and held for 10 min followed by final air-cooling to room temperature [16]. During the actual production process, laminar cooling was carried out after finishing the rolling process, and the cooling speed was close to 50 °C/s. Thus, the cooling rate of 50 °C/s was selected in this work. The critical partitioning time to finish carbon partitioning was calculated according to the kinetics model of carbon partitioning proposed in the literature [17]. The result indicates that it took about 6 min to finish carbon partitioning at 310 °C for the tested steel. Therefore, the longer isothermal holding time of 10 min was designed for completed partitioning.

After the thermal simulation experiments, the specimens were sampled along the central section perpendicular to the deformation direction. Firstly, the volume fraction and carbon concentration of RA after different treatments were measured by X-ray diffractometer (XRD, SmartLab SE, Tokyo, Japan) at a scanning speed of 1~2°/min under 40 KV, 40 mA, and Cu-Kα modes. The volume fraction of RA was obtained using the following Equation (1) [18]:

(1) $$V_{A}=\frac{1}{1+G(I_{\alpha }{\;/\;I}_{\gamma })}$$

where *V*_A_ represents the volume fraction of RA, I_α_ represents the cumulative strength of the ferrite peak, I_γ_ represents the cumulative strength of the austenite peak, and the corresponding G value for each peak pair was used as follows: 2.46, 1.32, and 1.78 for I_α(200)_/I_γ(200)_, I_α(200)_/I_γ(220)_, and I_α(200)_/I_γ(311)_, respectively; 1.21, 0.65, and 0.87 for I_α(211)_/I_γ(200)_, I_α(211)_/I_γ(220)_, and I_α(220)_/I_γ(311)_, respectively.

The carbon concentration in austenite was calculated by Equation (2) [19]:

(2) $$C_{\gamma }=(\alpha_{\gamma }-3.547)-0.046$$

where *C*_γ_ is the carbon concentration expressed as a percentage, and α_γ_ is the lattice parameter of austenite in Angstroms, estimated by Equation (3).

(3) $$\alpha_{\gamma }=\frac{\lambda \sqrt{h^{2}+k^{2}+l^{2}}}{2\sin\theta }$$

where λ is the radiation wavelength of the X-ray, (hkl) is the Miller indices, and θ is the Bragg angle.

According to the Williamson–Hall method [20,21], the dislocation density of martensite was measured by analyzing the (200) and (211) X-ray diffraction peaks, as expressed by Equation (4) [21]:

(4) $$\rho =\frac{14.4\varepsilon^{2}}{b^{2}}$$

where *ρ* is the dislocation density, *ε* is the micro strain, and b is the Burgers vector (0.247 nm). Considering the (200)α and (221)α peaks, the microstrain was calculated by Equation (5) [22]:

(5) $$\delta_{\mathrm{hkl}}\frac{\cos\theta_{\mathrm{hkl}}}{\lambda }=\frac{1}{D}+2\varepsilon \frac{\sin\theta_{\mathrm{hkl}}}{\lambda }$$

where δ_hkl_ is the physical broadening of the full width at half maximum (FWHM) of the diffraction peak, and *D* is the microcrystal size parameter.

The microstructure of the samples was observed by a field emission scanning electron microscope (FE-SEM, Nano 400, Stanford, CA, USA) after mechanical polishing and etching in 4% nital solution (Chemical Reagent Co., Ltd., Shanghai, China). The grain size and grain boundaries were measured by electron backscatter diffraction (EBSD, Oxford Symmetry, thermo Fisher Ltd., Waltham, MA, USA) with a scanning step of 0.1 μm at a voltage of 20 kV. A field emission transmission electron microscope (TEM, JEM-F200, Tokyo, Japan) was utilized to characterize the RA and other substructures. The mechanical properties were tested at room temperature using an Instron-3382 tensile tester (Instron Ltd. Instron, Norwood, MA, USA) with a strain rate of 0.001 s^−1^. The sub-size specimens with a thickness of 1.2 mm, gauge length of 8 mm, and gauge width of 1.3 mm were utilized in tensile tests.

## 3. Results and Discussion

### 3.1. Mechanical Properties

**Figure 2:**
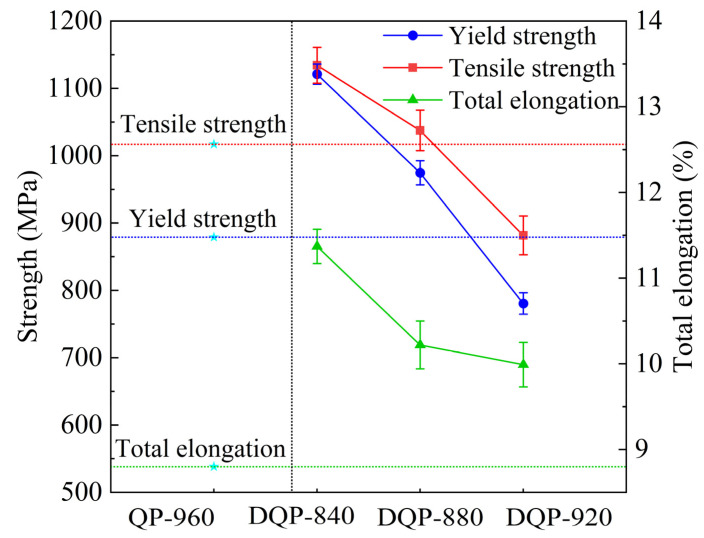
The stress-strain curve of Q&P [23] and DQ&P at different deformation temperatures. (Stars refer to the mechanical properties of Q&P steel).

In order to highlight the performance superiority of the DQ&P processing route, the mechanical properties of a Q&P steel with similar chemical compositions in the literature [23] are shown in Figure 2 for comparison. The regular Q&P process in the reference is one-step isothermal holding between Ms and Mf after austenitizing at 960 °C (QP−960) without deformation, and finally, the Q&P steel with a yield strength of 879 MPa, a tensile strength of 1017 MPa, as well as the total elongation of 8.0% were prepared. It was known that the product of ultimate tensile strength (UTS) and total elongation (TE) i.e., UTS × TE [24,25], which is an index of formability, is often used to compare the strength-ductility balance for different microstructures [25,26]. The formability index (UTS × TE) of DQ&P specimens (8.8~13.9 GPa·%) is better than that of regular Q&P steel (8.1 GPa·%). The results show that DQ&P steel can obtain higher comprehensive mechanical properties with respect to Q&P steel at similar compositions.

In addition, compared with the finishing rolling temperature of 920 °C (DQP−920), the yield strength increased by 194 MPa, the tensile strength increased by 156 MPa at the finishing rolling temperature of 880 °C (DQP−880), and the total elongation was basically unchanged. When the finishing rolling temperature is 840 °C (DQP−840), the yield strength increased by 340 MPa, the tensile strength increased by 252 MPa, and the total elongation increased by 1.4%. Therefore, with a decrease of finishing rolling temperature, the strength and total elongation increases.

### 3.2. Flow Behavior under Double-Passes Hot Deformation

**Figure 3:**
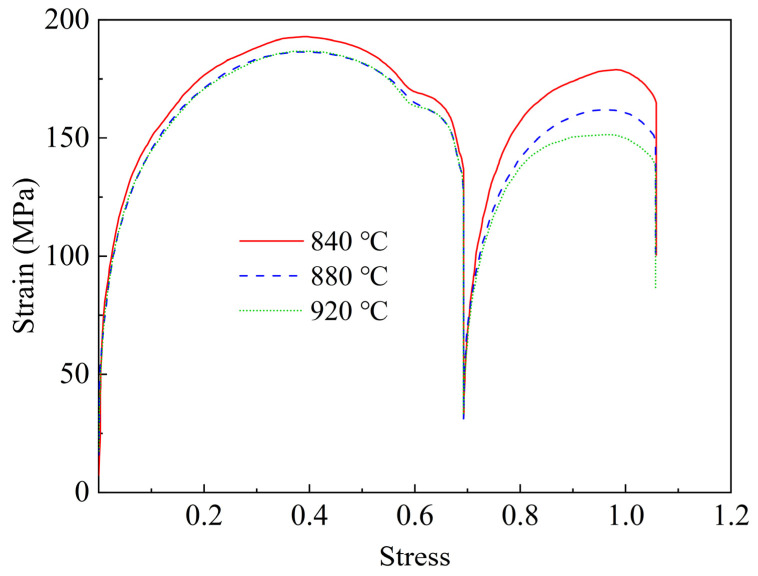
Flow curves of specimens at different rolling temperatures under double-pass hot deformation.

**Figure 4:**
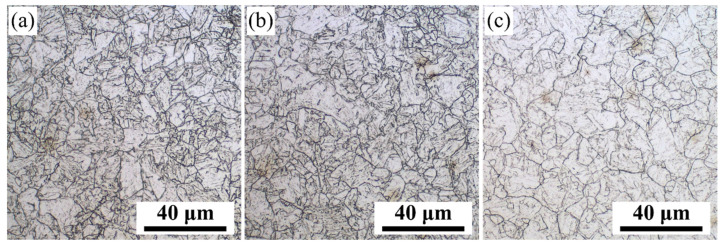
Original austenite grains of the tested steel at different finishing rolling temperatures: (**a**) 840 °C, (**b**) 880 °C, (**c**) 920 °C.

The stress-strain curve at different deformation temperatures during the hot deformation in the double-pass method is shown in Figure 3. The stress-strain curve of the first-pass deformation of rough rolling contains a work-hardening region and a stress peak, showing typical dynamic recrystallization behavior. When the strain reaches 0.7, compressive stress is unloaded. As the specimen was placed horizontally between two clamps, the stress of 31 MPa was maintained for fixed specimens. Then the flow behavior in the second-pass deformation of finish rolling exhibits apparent work hardening and strain softening at different deformation temperatures, which causes another peak stress [27]. The second non-recrystallization zone in Figure 3 is consistent with the characteristics of the industrial TMCP process. The result shows that the deformation resistance increases as the finishing temperature decreases, which can further refine the original austenite grains. The original austenite grain size was counted using the intercept method with Nano Measurer 1.2 software (Fuh Tan University, China). The original austenite microstructure at different finishing rolling temperatures is shown in Figure 4. To ensure the accuracy of the results, the average of several different regions of the microstructure was taken as the final result. The statistical results show that the average grain size of the original austenite was 12.18 μm, 10.89 μm, and 8.40 μm at 920 °C, 880 °C, and 840 °C, respectively. Therefore, it is seen from Figure 4 that the average grain size of the original austenite decreased by decreasing finishing rolling temperature. It is well known that the deformation applied in the single austenite region plays an important role in grain refinement [28]. As the finishing rolling temperature decreases, the thermal activation energy of the deformation of material increases, and the driving force of partial recrystallization decreases. At the same time, due to the density of defects, such as a large number of dislocations is greatly increased, the dynamic recovery effect is obviously weakened with decreasing finishing rolling temperature; that is, the grain deformation is obviously thinner [29]. Therefore, it can be concluded that enhancing the deformation work-hardening effect and decreasing both the dynamic recovery and recrystallization rate by decreasing the finishing rolling temperature, results in the obvious refinement of the original austenite grains of DQ&P steel at 840 °C [29,30]. 

### 3.3. Microstructure Characteristics

**Figure 5:**
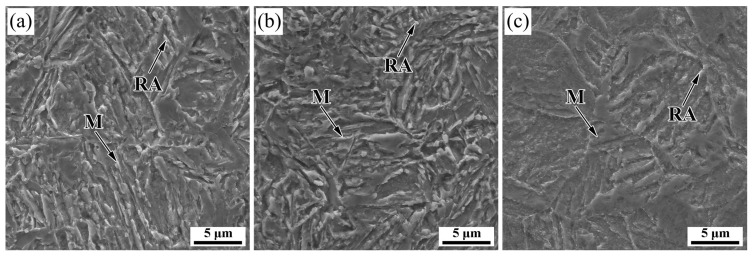
Microstructure of the tested steel at different finishing rolling temperatures: (**a**) 840 °C, (**b**) 880 °C, (**c**) 920 °C.

The SEM images in the central section along the deformation direction of the tested steel at different finishing rolling temperatures are shown in Figure 5. It is observed that the microstructure of all deformed specimens is mainly composed of martensite (M) and RA. The martensite presents a typical lath morphology. Compared with Q&P steel, the lath martensite (LM) is discontinuous, and the edges are serrated by applying deformation in the finishing rolling temperature region of austenite during the DQ&P process [15,31]. This is attributed to the high dislocation density in austenite caused by grain refinement due to austenite deformation, which together inhibits the growth of the martensite laths, resulting in the distortion or rupture of the martensite laths and jagged edges during the DQ&P process. In addition, some fresh martensite (FM) with smooth surfaces and white boundaries can be observed in the deformed specimens, which are formed by the shear transformation of unstable, untransformed austenite in the final cooling process [32].

**Figure 6:**
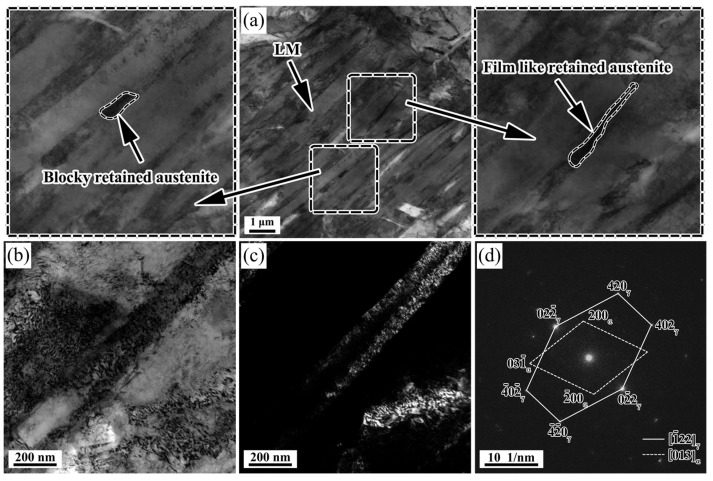
Specimens at 840 °C: (**a**) TEM micromorphology, (**b**) bright field image, (**c**) dark field image, and (**d**) related selected area electron diffraction pattern of martensite and RA.

Further characterization of RA was observed using TEM. The microscopic morphology of the specimen at 840 °C is shown in Figure 6. It is seen from Figure 6a that the microstructure mainly consists of LM and RA, and the RA exists in the form of film and block between the martensite laths. Furthermore, the LM has a high dislocation density, and the corresponding yield strength would be high, which will inhibit the further secondary martensite transformation during the final cooling process. Because the surrounding matrix needs to be deformed to adapt to the volume expansion caused by martensite transformation, martensite with high-yield stresses can “shield” austenite from external loads during the deformation process [33]. In addition, Figure 6b–d shows the bright and dark field images and selected area electron diffraction patterns of martensite and RA after finishing rolling at 840 °C. It can be seen from the selected area electron diffraction pattern in Figure 6d that the diffraction spots include both the bcc structure of martensite and the fcc structure of austenite, which further confirms the existence of RA.

**Figure 7:**
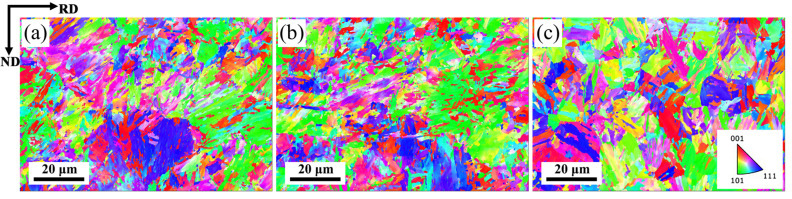
EBSD inverse pole figure (IPF) at different finishing rolling temperatures: (**a**) 840 °C, (**b**) 880 °C, (**c**) 920 °C.

**Figure 8:**
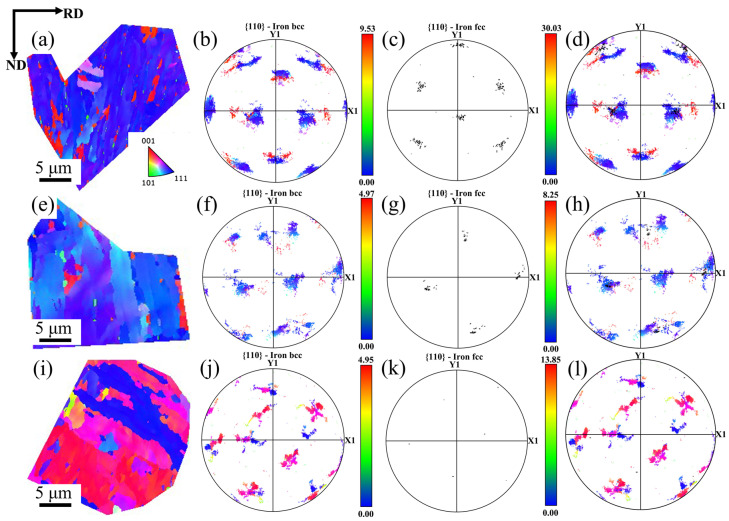
(**a**–**d**) 840 °C, (**e**–**h**) 880 °C, and (**i**–**l**) 920 °C: (**a**) the pole figure; (**b**) the pole figure of {110} plane of bcc structure; (**c**) the pole figure of {111} plane of fcc structure; (**d**) the superimposed pole figure of {110} bcc plane and {111} fcc plane.

EBSD inverse pole figures (IPFs) of the specimens are shown in Figure 7. It indicates that most of the bcc structure is aggregated in the {101} and{111} orientations, indicating that most of the martensite grains exist in the same orientation laths. The IPF and pole figures of the specimens are shown in Figure 8. It can be seen that the martensite variation of the specimens exhibits obvious differences compared with the regular Q&P process, by which the martensite and austenite keep the typical Kurdjumov–Sachs (K–S) relationship [34]. As the finishing rolling temperature increases, the deviation of the K–S orientation relationship between the matrix bcc phase and the parent phase austenite gradually increases [35].

**Figure 9:**
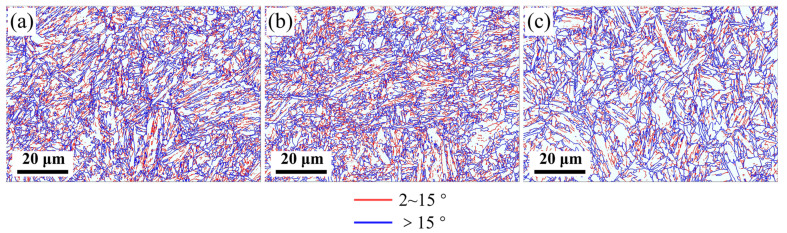
Grain boundary distribution of the specimens at different finishing rolling temperatures: (**a**) 840 °C, (**b**) 880 °C, (**c**) 920 °C (red represents 2 to 15°, blue represents equal to or larger than 15°).

The grain boundary maps in Figure 9 shown the high-angle grain boundaries (HAGBs, >15°) in blue and the low-angle grain boundaries (LAGBs, 2−15°) in red. As a result, the length of the HAGB increases from 7.46 mm at 920 °C to 8.46 mm at 880 °C, and then further increases to 9.06 mm at 840 °C as the finishing rolling temperature decreases.

**Figure 10:**
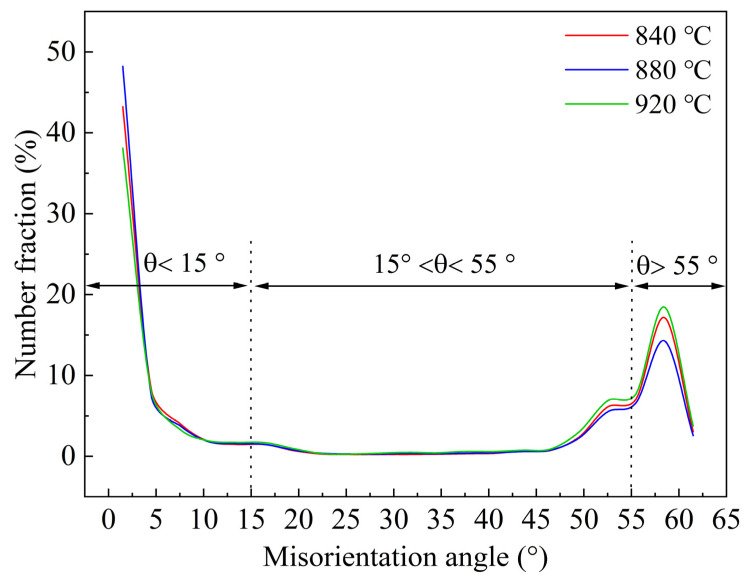
Grain orientation distribution map at different finishing rolling temperatures.

Figure 10 shows the distribution of misorientation angles of the specimens. It can be seen that the misorientation angles of the samples at different finishing rolling temperatures are mainly less than 10° and greater than 50°, and three peak values of misorientation in different directions are detected, i.e., 16.5°, 52.5°, and 58.5° [19]. These peaks are the result of different variants of the K–S orientation relationship [36]. There are ten possible orientations between the two martensitic substructures, i.e., 10.5°, 14.9°, 20.6°, 21.1°, 47.1°, 49.5°, 50.5°, 51.7°, 57.2°, and 60° [37]. Therefore, there is a deviation between the actual orientation and the ideal orientation of martensite, and the three orientation peaks of 16.5°, 52.5°, and 58.5° correspond to the boundaries of blocks and packets [36,37,38], which is consistent with the microstructure of the specimens at all finishing rolling temperatures consisting of the mixture of LM and RA [19,36]. In addition, the absence of a misorientation boundary between 20° and 45° coincides well with the ideal misorientation between the K–S variants [36,37,38].

**Figure 11:**
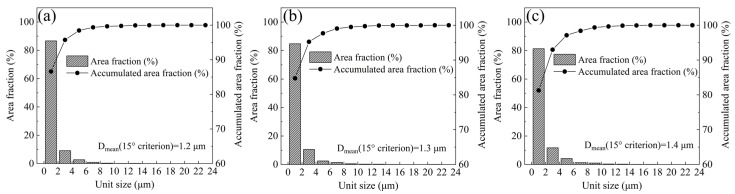
(**a**) 840 °C, (**b**) 880 °C, and (**c**) 920 °C at 2° and 15° misorientation, respectively.

According to the average equivalent circle diameter, the crystallographic unit sizes of 15° misorientation were measured by EBSD. The number of grains in the samples are sufficiently large (approx. 3000) to guarantee the measurement results are accurate. According to the statistical results in Figure 11, based on the 15° misorientation standard, the average equivalent circle diameters are 1.2 μm, 1.3 μm, and 1.4 μm, respectively. The statistical results show that the average equivalent circle diameter at 840 °C is below that at 880 °C and 920 °C. Due to many martensite laths, blocks and packets are high-angle grain boundaries [39]; it is confirmed that a refined microstructure can be obtained by finishing rolling at 840 °C.

In summary, the refined original austenite grains promote the refinement of the LM structure at room temperature. This is due to the fact that the martensitic transformation occurring in fine-grained austenite requires a large driving force and nucleation energy after quenching. Compared to the nucleation of relative coarse-grained austenite at 920 °C and 880 °C, finishing rolling at 840 °C provides more chemical-free energy to drive fine-grained martensitic transformation [40]. Therefore, the deformation inhibits the grain growth, resulting in a decrease in the supercooling degree and limiting the growth of LM, thereby refining the lath martensite at 840 °C. Therefore, the finer LM improved the strength of the tested steel. Early studies have shown that the {111} oriented grains have high stability and gradually transfer to the {101} oriented polar axis during the tensile deformation process, which is beneficial to the plastic properties [41]. According to the statistical results, the {111} orientation increases with temperature, accounting for about 12.2%, 12.8%, and 17.1%, respectively. In addition, the HAGB length at 840 °C is higher than that at 880 and 920 °C, which is beneficial to inhibit crack propagation and improve ductility [4,42]. Therefore, the tensile properties after final rolling at 840 °C are better than at 880 °C and 920 °C.

### 3.4. RA Analysis

**Figure 12:**
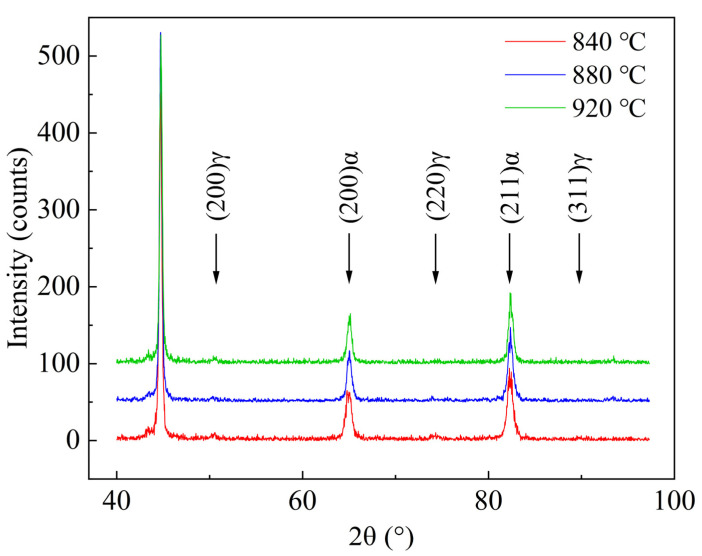
XRD diffraction pattern at different finishing rolling temperatures.

The RA content was detected by X-ray diffractometer (XRD). The experimental results are shown in Figure 12. It can be judged that the room-temperature phase is mainly bcc crystal structure and fcc crystal structure, corresponding to ferrite or martensite and RA, respectively.

According to Formulas (1)–(5), it is calculated that the volume fraction of RA, carbon concentration, and the dislocation density in martensite are shown in Table 2.

**Figure 13:**
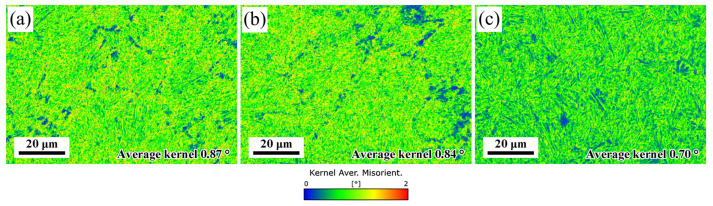
Kernel average misorientation at different finishing rolling temperatures: (**a**) 840 °C, (**b**) 880 °C, (**c**) 920 °C.

It is seen from Table 2 that the volume fraction of RA and dislocation density in martensite increase as finishing rolling temperature decreases. The dislocation density can also be represented by kernel average misorientation (KAM) maps, as given in Figure 13 [43]. It is well known that the higher the KAM value, the higher the dislocation density in the microstructure. It is seen that the average KAM value increases as the finishing rolling temperature decreases, indicting the same change tendency of dislocation density as the XRD results. As mentioned above, the finer original austenite grain is formed at 840 °C, which may significantly reduce the undercooling degree [44], resulting in a decrease in the nucleation driving force of the martensitic transformation [45], thereby reducing the martensite amount and increasing the fraction of untransformed austenite. In addition, decreasing the austenite grain size is one of the main reasons for improving austenite stability [35]. The size of the austenite grain is smaller, the interfacial area between the grain and its surrounding martensitic phase is larger per unit austenite volume fraction [31]. This provides more diffusion channels to absorb carbon atoms from martensite in unit volume. At the same time, the energy stored in the austenite during deformation has a more significant effect on the martensitic transformation and influences the carbon partitioning during the partitioning process. The applied deformation reduces the original austenite grain size while generating a large number of high-density dislocations, which also provide favorable diffusion channels for carbon distribution. Obviously, finer grain size and higher dislocation density generated by deformation improve the stability of RA.

In summary, the tensile test results show that deformation at a finishing rolling temperature lower than 880 °C can improve the tensile properties. RA can significantly improve the mechanical properties of the specimens through the TRIP effect [8]. In addition to the TRIP effect, the finer lath martensite microstructure in the 840 °C samples also increase the tensile strength. Early studies have shown that the RA content and its stability have a substantial influence on the formability of the evolved structure [35,46]. The thermal stability of RA is influenced by several factors such as grain size, carbon concentration, and morphology of RA [35,47]. Therefore, the evolution of mechanical properties is discussed according to the original austenite grain size, volume fraction, carbon concentration, and morphology of RA. As mentioned above, the stability of RA can be improved by the refinement of original austenite grains. RA was found to be present in the form of films and blocks between the martensite laths by TEM analysis (Figure 6). It is documented in the literature that the two morphologies have different stability during stretching to provide a continuous TRIP effect [48]. The film-like RA has a higher phase transformation stability due to its lower area and longer length, which are more easily enriched from carbon atoms [49]. At the same time, blocky RA promotes work hardening through the TRIP effect in the early stage of deformation, while a small part of blocky RA plays a role in coordinating deformation during the whole deformation process. The carbon concentration of RA at 840 °C final rolling is higher than that at 920 °C and 880 °C final rolling according to the XRD results (Table 2), which can further indicate that RA has higher stability at 840 °C final rolling. In addition, according to the EBSD test results (Figure 11), the LM can be refined by finishing rolling at 840 °C. The stable RA undergoes a TRIP effect during plastic deformation, transforming into a high-strength martensite, inhibiting the instability of plastic deformation and increasing uniform elongation (Figure 3), thereby increasing both the strength and plasticity of the steels [12]. Therefore, it is reasonable to obtain the better total elongation of 11.7% and the best tensile strength of 1134 MPa after finishing rolling at 840 °C.

## 4. Conclusions

In this paper, the microstructure and properties of a DQ&P steel at different final rolling temperatures are systematically investigated. The following conclusions can be drawn:

(1) Compared with Q&P steel with similar compositions, relatively excellent comprehensive mechanical properties can be obtained by DQ&P processes. The strength and total elongation of a low-carbon DQ&P steel were significantly improved after finishing rolling at 840 °C.
(2) With the decrease of finishing rolling temperature from 920 °C to 840 °C, the strength and total elongation increased. High-yield strength of 1121 MPa, high tensile strength of 1134 MPa, and 11.7% total elongation at 840 °C were achieved.
(3) With the decrease of finishing temperature, the lath martensite became finer, and the RA fraction increased. Grain refinement and higher dislocation density contributed to carbon partitioning, leading to the higher stability of RA at a lower finishing temperature.

## Figures and Tables

**Table 1:** The chemical composition of the tested steel (wt.%).

C	Si	Mn	Ti	N	Al	S	P
0.21	1.8	2.03	0.017	0.004	0.04	0.002	0.015

**Table 2:** The volume fraction of RA, carbon concentration, and the dislocation density in martensite at different finishing rolling temperatures.

Temperature (°C)	840	880	920
Volume fraction of retained austenite (%)	5.90	4.30	3.01
Carbon content (%)	1.51	1.48	1.41
Dislocation density (m^−2^)	2.06 × 10^16^	1.41 × 10^16^	1.23 × 10^16^
